# Covid-19 vaccination programme effectiveness against SARS-CoV-2 related infections, hospital admissions and deaths in the Apulia region of Italy: a one-year retrospective cohort study

**DOI:** 10.1038/s41598-022-23235-4

**Published:** 2022-11-03

**Authors:** Tobias Homan, Sara Mazzilli, Antonio Chieti, Alessandra Musa, Adam Roth, Francesca Fortunato, Lucia Bisceglia, Rosa Prato, Pier Luigi Lopalco, Domenico Martinelli

**Affiliations:** 1 Hygiene Unit, Policlinico Foggia Hospital, Department of Medical and Surgical Sciences, University of Foggia, Ospedale Colonnello D’Avanzo, Viale Degli Aviatori, 2, 71122 Foggia, Italy; 2 ECDC Fellowship Programme, Field Epidemiology Path (EPIET), European Centre for Disease Prevention and Control (ECDC), Solna, Sweden; 3 Scuola Normale Superiore - Pisa, Toscana, Italy; 4 Strategic Regional Health and Social Agency of Puglia (AReSS Puglia), Bari, Italy; 5 European Centre for Disease Prevention and Control, Solna, Sweden; 6 Department of Biological and Environmental Sciences and Technology, University of Salento, Lecce, Italy

**Keywords:** Vaccines, Public health, Viral infection

## Abstract

Studies reporting vaccine effectiveness against COVID-19 outcomes concentrate mainly on estimates of one single type of vaccine and variant, seldom considering waning effects. We aimed to estimate the effectiveness of the overall COVID-19 vaccination programme implemented in the Apulia region of Italy at preventing SARS-CoV-2 infections, COVID-19-related hospital admissions and deaths during alpha and delta variant dominant periods. We conducted a retrospective cohort study using electronic health records of persons 16 years and older resident in the Apulia region, assessing the effectiveness of the combined use of BNT162b2, mRNA-1273, ChAdOx1-S and Ad26.COV2.S vaccines against confirmed COVID-19 infections, hospitalisations and deaths, for fully and partially vaccinated persons as well as by time since vaccination and variants. Cox regression models yielding hazard ratios were used to calculate the overall vaccination programme effectiveness. From 1 January to 1 December 2021, we included 3,530,967 eligible persons in the cohort, of whom 2,770,299 were fully vaccinated and 158,313 were COVID-19 positive at the end of the study period. The effectiveness of the programme over the entire study period for fully vaccinated persons against COVID-19 infection, hospitalisation and death were 87.69% (CI95% 87.73–88.18), 94.08% (93.58–94.54) and 95.95% (CI95% 95.26–96.54), respectively. The effectiveness against COVID-19 infection of fully vaccinated subjects during the alpha and delta period was respectively 88.20% (CI95% 87.60–99.78) and 59.31% (CI95% 57.91–60.67), against hospitalisation 93.89% (CI95% 92.67–94.90) and 88.32% (CI95% 86.50–89.90) and against death 93.83% (CI95% 91.65–95.45) and 85.91 (CI95% 79.98–90.09). The waning effects of the programme regarding COVID-19 infection during the delta period were stronger than for alpha, with 75.85% (CI95% 74.38–77.24) effectiveness after 1–2 months and 8.35% (CI95% 3.45–13.01) after 5–6 months after full vaccination. The effectiveness against hospitalisation and death during the delta period waned rapidly and at 7–8-months after the full vaccination respectively decreased to 27.67% (CI95% 7.48–43.45) and 48.47 (CI95% 53.97–34.82). Our study suggests that the COVID-19 vaccination program in Apulia was strongly protective against COVID-19 infection, hospitalisation, and death due to alpha as well as delta variants, although its effectiveness is reduced over time.

## Introduction

As of January 10, 2022, the severe acute respiratory syndrome coronavirus 2 (SARS-CoV-2) pandemic has resulted in more than 308 million cases and more than 5,5 million deaths worldwide^1^. Within one year, five COVID-19 vaccine candidates have been approved by the European Medicines Agency^2^ as well as the Italian medicines agency (AIFA)^3^, with several more in the development pipeline. The approved vaccines as originally shown in trials suggested to be highly efficacious against moderate-to-severe disease among adults, with an efficacy of 95% for the BNT162b2 (Pfizer–BioNTech) vaccine^4^, an efficacy of 94% for the mRNA-1273 (Moderna) vaccine^5^, an efficacy of 70% for the ChAdOx1-S (Astra Zeneca) vaccine^6^, an efficacy of 72% for the Ad26.COV2.S (Johnson & Johnson–Janssen) vaccine^7^, and an efficacy of 90% for the NVX-CoV2373 (Novavax) vaccine^8^. However, the end points of those trials were evaluated at a relatively short follow-up of 14 to 28 days after series completion, with notable limitations of sample size, restrictive inclusion criteria and during a period when the circulating strains were less transmissible than the currently circulating variants of SARS-CoV-2^9^.

Since the introduction of the approved vaccines, various real-world studies have reported on the effectiveness of partial and full vaccination against COVID-19 related infection, hospitalisation, and death^10–12^. Nevertheless, although initially the effectiveness was found to be closely reflecting the results of efficacy trials, extended trial follow-up and an increasing number of real-world effectiveness studies have begun to document declines in vaccination effectiveness (VE) for some outcomes and in some populations during 2021^12,13^. An important reason for this reduction is suggested to be due to the emergence of new strains of the original COVID-19 virus found in 2019^13–15^. After the initial “wild type” variant (alpha: B.1.1.7), four main variants of concerns have been identified to date (Beta: B.1.351, Gamma: P.1, Delta: B.1.617.2 and Omicron: B.1.1.529)^9^. Although data suggests that reduced sensitivity of variants to neutralising antibodies occurs^16^, substantial levels remain^17^. However, the main driver for the reduced effectiveness of vaccine programmes is related to the waning of vaccine immunity^18,19^.

Understanding the impact of these factors, namely the emergence of variants and waning immunity, in relation to the success of vaccination programmes is of paramount importance. Post authorization analyses meet the urgent need to evaluate the magnitude and sources of changes in the effectiveness of COVID-19 vaccines implemented through different vaccination strategies and across diverse populations with a wide range of coexisting conditions. Ultimately with the aim to develop vaccine recommendations and inform public health policy. In this study, we assessed the effectiveness of the COVID-19 vaccination programme implemented in the Apulia region of Italy in preventing SARS-CoV-2 infections, COVID-19-related hospital admissions and deaths. It has been almost two years since mass vaccination campaigns using newly approved vaccines against SARS-CoV-2 started in the Apulia region. The availability of vaccination data from nearly four million people over almost two years of vaccination programme allows a comprehensive narrative about its effects on COVID-19 related outcomes.

## Methods

### Study design and data sources

We conducted a retrospective cohort study by analysing electronic health records from the Apulia region health-care information system. We assessed the effectiveness of the combined use of four vaccines (BNT162b2, mRNA-1273, ChAdOx1-S and Ad26.COV2.S) in the immunization programme against SARS-CoV-2 infections, COVID-19-related hospital admissions and deaths. We also performed the analysis separately for mRNA vaccines (BNT162b2 and mRNA-1273) and viral vector vaccines (ChAdOx1-S and Ad26.COV2.S). The study period ran from 1 January to 1 December 2021.

A total of 4.09 million inhabitants were registered in the Apulia region as of December 1, 2021. In the study cohort, we included all residents aged ≥ 16 years. We excluded from the analysis individuals who had tested positive for SARS-CoV-2 infection before January 1, 2021. Moreover, people who died for other causes than COVID-19 or moved out of the region during the study period ceased to be in the study cohort at the date of death or at the date of moving. Electronic health records integrated clinical data including diagnostic, laboratory, hospitalisation, and vaccination history information across all settings of care. A comorbidity assessment was associated with the study participants through a medical vulnerability index taking values from 1 to 100. As extensively described elsewhere^20^, the index was based on demographic (age and sex) and clinical (29 conditions and diseases) predictors of the COVID-19 severity. According to the index, our study population was categorized into 3 groups: not fragile/at low level of fragility (index value 0–39: risk of occurrence of severe/fatal forms of COVID-19 < 0.1%), fragile (index value 40–59: risk between 0.1 and 1%) and very fragile (index value ≥ 60: risk more than 1%). We considered as vaccine doses administered those recorded in the regional Immunization Information System. If a record of a second dose was found without a record reporting the first dose, we considered the registered dose to be the first. The study followed the guidelines of the STROBE (STrengthening the Reporting of Observational studies in Epidemiology). The protocol for this study was reviewed by the Apulia region Health Authority. As this study constituted public health surveillance, ethical approval from institutional review board was not required. All data were provided and analysed anonymously.

### Study context

The Apulia region is located in southeast Italy and consists of six administrative divisions, named provinces. In Italy, the Ministry of Health led vaccination programme planning. The regional authorities are in charge of organizing and implementing the new vaccination strategies at the local level. The COVID-19 vaccination programme in Italy began on December 27, 2020, and it was at the start targeted at health care workers, residents in nursing homes, and medical risk groups. Gradually, descending by 10-year steps and extending to other vulnerable groups, over the first six months of 2021 eventually all population over 16 years were called to receive full anti-COVID-19 immunization. The BNT162b2 vaccine was available since 27 December 2020, however initially the number of doses were extremely limited. The mRNA-1273 and ChAdOx1-S vaccines were subsequently delivered during the next months. In the case of the ChAdOx1-S vaccine, it was administered mainly to some professional risk groups, such as security forces workers and school staff. By April 2021 small numbers of the Ad26.COV2.S vaccines started to be administered in the Apulia region. In March and April ChAdOx1-S and Ad26.COV2.S were scrutinised for adverse effects and temporarily suspended but their administration occurred mostly until mid-August. With the worldwide production of vaccines substantially increasing, the majority of the vaccines administered in the Apulia region since May were the BNT162b2 vaccine^21^.

### Study groups and outcomes

Full vaccination status was defined as having received a single dose of Ad26.COV2.S or two doses of BNT162b2 or mRNA-1273 or ChAdOx1-S or a combination of them from seven days after vaccination. Individuals were defined partially vaccinated if they received only one dose of BNT162b2 or mRNA-1273 or ChAdOx1-S from 7 days or more or if they received two doses of COVID-19 vaccine for less than 7 days. Individuals were considered unvaccinated until seven days after administering of their first dose of vaccine, or until censoring at disenrollment or death. A person was classified as exposed if one or two doses of vaccine were administered between January 1 and December 1 2021, with maximum follow-up time censored until December 1, 2021. Persons that received a third/booster dose were censored from the date of that dose.

Outcomes comprised SARS-CoV-2 infection, defined as a positive real-time PCR test result or as a positive third-generation antigen test result obtained from any public or private facility in the Apulia region, COVID-19 related hospitalisation and death. Every health care provider in the Apulia region is obligated to submit SARS-CoV-2 infection related results to the regional health authorities. According to surveillance data of Apulia region, nearly 90% of cases between January 1 and July 1 2021 were caused by the alpha variant. In June, 20% of SARS-CoV-2 sequenced resulted to be delta variant increasing up to the 80% in July, while as of December 1, over the 90% of cases were caused by delta^22^. Since in the Apulia region no population-wide information on sequencing results was available, we used the evidence above-mentioned to determine the variant for SARS-CoV-2 causing the infection. Hence, all COVID-19 cases between January and June 2021 were considered caused by alpha variant, while from July onwards all cases were considered caused by delta variant. Hospital admissions were included if COVID-19 was the main cause for the hospitalisation. A COVID-19 related death was defined as a death with a positive SARS-CoV-2 test that was conducted in the 28 days prior to death.

### Statistical analysis

Descriptive statistics reported demographic variables and a medical vulnerability index. We presented the cumulative number of fully and partially vaccinated persons over time in the Apulia region by sex, age category (16–44 years, 45–64 years, and 65+ years), province and fragility index (not fragile, fragile, very fragile), as well as the number of fully vaccinated persons per type of vaccine and by age at the end of the study period. Subsequently, the number of infections by vaccination status was presented. Person-time for unvaccinated persons was comprised of follow-up time for those vaccinated until censoring, partial or full vaccination, in addition to the time contributed by those never vaccinated.

In the primary analysis we compared infection, hospitalisation, and death rates over the whole study period between unvaccinated and fully vaccinated as well as between unvaccinated and partially vaccinated. We performed this analysis by the whole cohort as well as by age category (16–44, 45–64, 65 +). Fully vaccinated persons were further analysed bi-monthly by time since vaccination (0 to 2 months, 3 to 4, 5 to 6, 7 to 8 months and 9 to 10 months) and by SARS-CoV-2 variant (alpha and delta). The calculation of follow-up time and COVID-19 outcomes were computed from seven days after the final vaccination dose. The follow-up time and COVID-19 outcomes of the unvaccinated group were kept constant to the full study period. Additionally, analyses were performed on time since infection by different COVID-19 virus variants. Time since vaccination for alpha cases was never more than 5.5 months (mid-January the first person was fully vaccinated), and therefore estimates were reported on a one-monthly basis. We allowed follow-up time for vaccinated with a COVID-19 event in the delta period to start before 1 July, whereas for the unvaccinated group follow-up time started from the delta period. Additionally to these analyses including all administered vaccine products, we reported the undifferentiated analyses also separately for mRNA vaccines (BNT162b2 and mRNA-1273) and viral vector vaccines (Ad26.COV2.S and ChAdOx1-S).

For our analyses we used Cox hazard regression models yielding hazard ratios (HRs) with 95% confidence intervals (CI95%). For all models, effectiveness of the combined use of four vaccines and mRNA/viral vector vaccines was computed as 1-HR, multiplied by 100. We added covariates to the unadjusted models to derive estimates in adjusted models comprising of calendar week (account for progressive vaccine eligibility, testing practices, non-pharmaceutical interventions, lockdown requirements, disease activity, and COVID-19 treatment changed over time), sex, age (per five years), province, week of infection (account for varying infection rates over time) and a fragility index.

We used RStudio (RStudio Desktop 2021.09.1 + 372, Integrated Development for R. PBC, Boston) for all analyses, including the survminer package for the analysis of survival data.

### Ethics approval

As this study was conducted within the public health surveillance programme established by the Ministry of Health, ethical approval was not required. All procedures contributing to this work complied with the ethical standards of the relevant national and institutional committees on human experimentation and with the Helsinki Declaration of 1975, as revised in 2008. Moreover, according to the Italian regulation (DETERMINAZIONE AIFA—20 marzo 2008, GU n. 76 del 31-3-2008), this retrospective epidemiological study had only to be notified to the Regional Public Health Authority for the nature of the study itself. Informed consent was not obtained from participants because surveillance data and molecular and antigenic testing were retrieved and analysed anonymously.

## Results

### Population characteristics

From 1 January to 1 December 2021, 3,530,967 individuals were included in the study cohort. The 51.72% of participants were female and 48.27% were male. The median age was 51.6 years old. Following the personal medical vulnerability index, 91.20% of the study participants were not fragile, 7.94% were fragile and 0.86% were very fragile.

**Figure 1:**
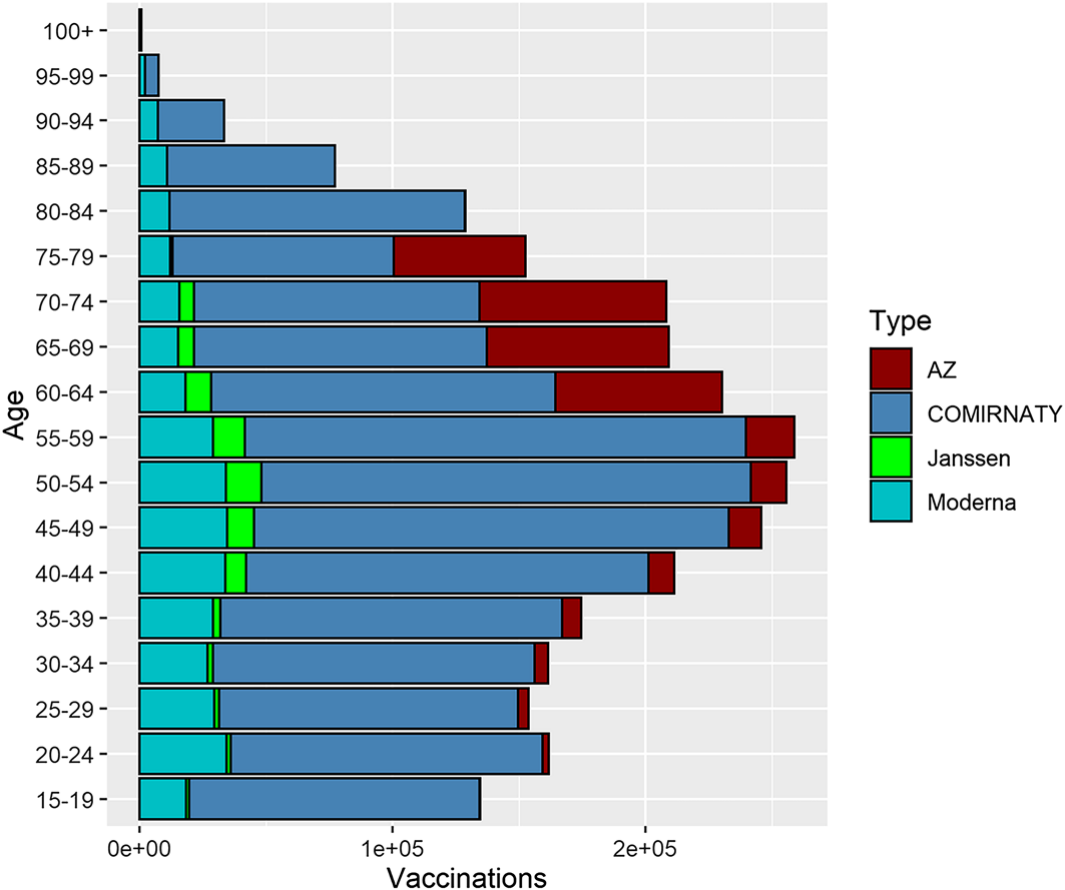
The number of fully vaccinated persons by age at the end of the study period. Apulia region, Italy, Jan 1–Dec 1, 2021.

**Figure 2:**
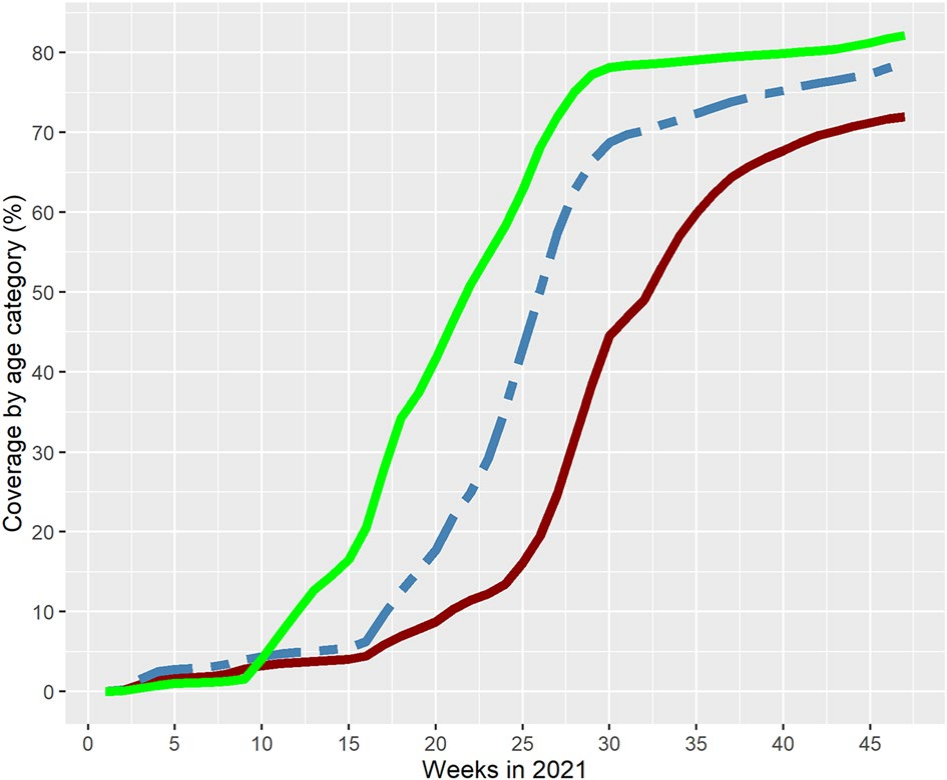
Cumulative proportion (coverage) of fully vaccinated persons by age categories (green line = 65 + years, blue line = 45–64 years, red line = 16–44 years) over time. Apulia region, Italy, Jan 1–Dec 1, 2021.

At the end of the study period 78.46% were fully vaccinated and 5.18% were partially vaccinated. A total of 1,997,940 (72.12%) individuals received 2 doses of BNT162b2, 373,713 (13.49%) 2 doses of mRNA-1273, 310,551 (11.21%) 2 doses of ChAdOx1-S, and 57,068 (2.06%) 1 dose of Ad26.COV2.S (Fig. 1). The 1.12% of double fully vaccinated persons received a mix of one of BNT162b2, mRNA-1273 and ChAdOx1-S vaccines. The cumulative proportion of fully vaccinated persons by age categories over time was showed in Fig. 2. Further details on demographic characteristics of the study population were showed in Table 1.

**Table 1:** Number and proportion of fully/partially vaccinated persons, by sex, category of age, and fragility index, and of COVID-19 cases, by calendar month and virus variant, included in the cohort at the end of the study period. Apulia region, Italy, Jan 1–Dec 1, 2021.

Study cohort	Unvaccinated (N. 577,854)	Partially vaccinated* (N. 182,814)		Fully vaccinated (N. 2,770,299)		Total population (N. 3,530,967)
	N	N	*%*	N	*%*	N
**Sex**						
F	285,265	92,092	5.04	1,449,063	79.34	1,826,420
M	292,589	90,722	5.32	1,321,236	77.51	1,704,547
**Age category, years**						
16–44	272,340	90,184	6.71	982,374	73.04	1,344,898
45–64	189,484	60,485	4.93	977,668	79.64	1,227,637
65 +	116,030	32,145	3.35	810,257	84.54	958,432
Fragility index**						
Not fragile	562,345	170,790	5.30	2,486,917	77.23	3,220,052
Fragile	13,986	10,897	3.88	255,625	91.13	280,508
Very fragile	1523	1127	3.71	27,757	91.28	30,407

COVID-19 cases	Unvaccinated (N. 138,441)	Partially vaccinated* (N. 7799)		Fully vaccinated (N. 12,073)		Total infection (N. 158,313)
	N	N	*%*	N	*%*	N
**Coverage by month of infection**						
January	3078	17,270	64.66	6362	23.82	26,710
February	2437	16,187	77.03	2389	11.37	21,013
March	5292	33,214	79.87	3079	7.40	41,585
April	4788	28,327	79.08	2704	7.55	35,819
May	1956	8361	71.89	1314	11.30	11,631
June	727	1070	49.56	362	16.77	2159
July	999	619	28.42	560	25.71	2178
August	2491	934	16.33	2296	40.13	5721
September	1271	249	7.29	1897	55.52	3417
October	753	114	3.64	2264	72.31	3131
November	962	113	2.28	3874	78.28	4949
**SARS-CoV-2 variant**						
Alpha	131,585	6107	11.41	1589	4.38	139,281
Delta	6856	1692	8.89	10,484	55.09	19,032

Individuals who deceased, moved out of the region, or had tested positive for SARS-CoV-2 infection before Jan 1, 2021, were excluded from this analysis.

*Persons were considered partially vaccinated if they received a single dose with 7 days or more after the first dose or when persons received two doses with less than 7 days after the second dose. After 7 days of receiving one dose of Ad26.COV2.S, persons were considered to be fully vaccinated.

**A person’s fragility was calculated on basis of an index derived from a list of scores on different health outcomes, see the methods section for details. The index is divided into 3 categories previously used by Italian health authorities.

A total of 158,313 persons were infected with SARS-COV-2, among whom 12,543 (7.92%) were admitted to hospital and 3658 (2.31%) deceased (SI Table 1) during the 11 months of study. 139,281 positive cases occurred during the alpha variant period (1 January–30 June) whereas the number of cases during the delta variant period (1 June–30 November) was 19,032 (SI Table 1).

### Overall vaccination programme effectiveness

**Figure 3:**
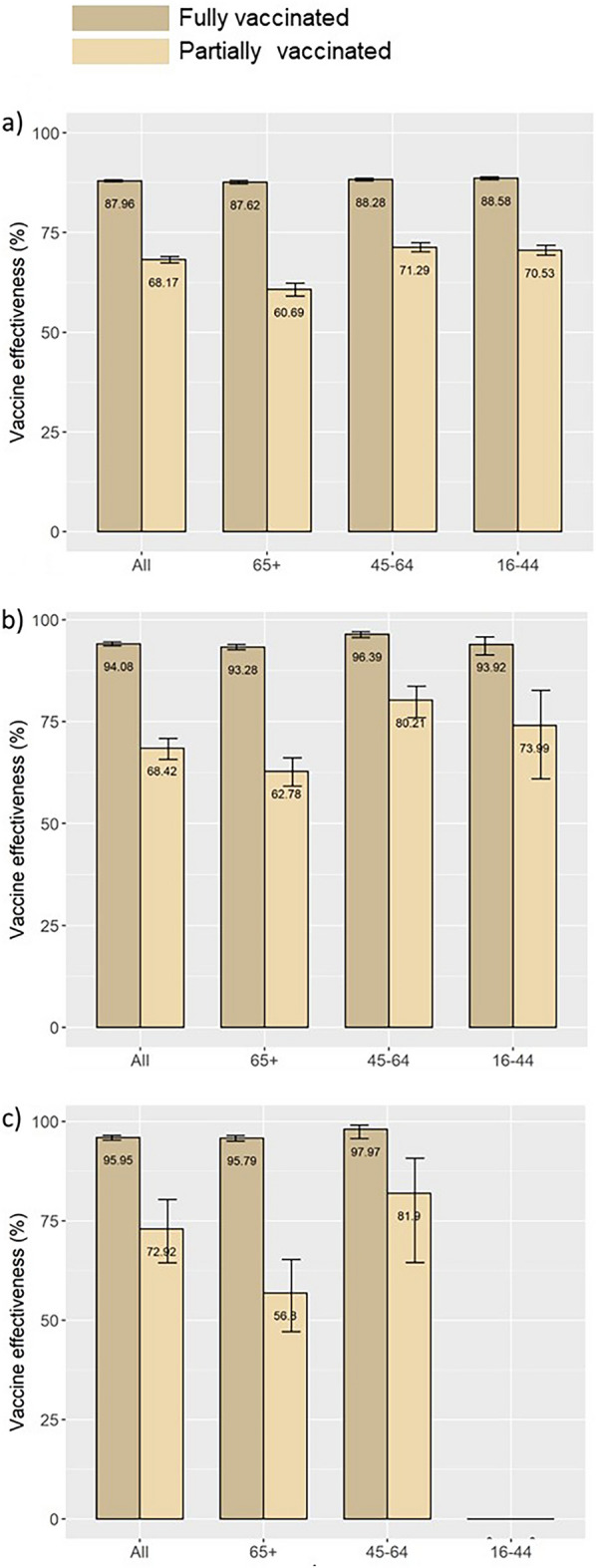
Adjusted effectiveness (estimates of COX models) of the vaccination programme against COVID-19 (**a**) infection, (**b**) hospitalisation, (**c**) death, by vaccination status and age category. Apulia region, Italy, Jan 1–Dec, 2021.

For the total cohort, the adjusted effectiveness of the COVID-19 vaccination programme against infections was 87.96% (CI95% 87.73–88.18) for individuals who were fully vaccinated and 68.17% (CI95% 67.40–69.93) for partially vaccinated persons (Fig. 3; SI Table 3a). The effectiveness against hospitalisation for fully vaccinated was 94.08% (CI95% 93.58–94.54) and 68.42% (CI95% 65.71–70.91) for partially vaccinated while the effectiveness against death was 95.95% (CI95% 95.26–96.54) for fully vaccinated and 72.92% (CI95% 64.39–80.35) for those partially vaccinated (Fig. 3; SI Table 3a).

The adjusted effectiveness of full vaccination against infection was similar for the three age groups reported: 88.58% (CI95% 88.20–88.94) for individuals between 16 and 44 years, 88.28% (CI95% 87.92–88.63) between 45 and 64 years, and 87.62% (CI95% 87.18–88.05) for those older than 64 years. The results were homogeneous in the three age groups also for the effectiveness against hospitalisation (age group 16–44: 93.92%, CI95% 91.30–95.75; age group 45–64: 96.39%, CI95% 95.61–97.03; age group > 64: 93.28%, CI95% 92.63–93.97) and death (age group 45–64: 97.97%, CI95% 95.71–99.04; age group > 64: 95.79%, CI95% 95.05–96.42) (Fig. 3; SI Table 3b–d).

**Figure 4:**
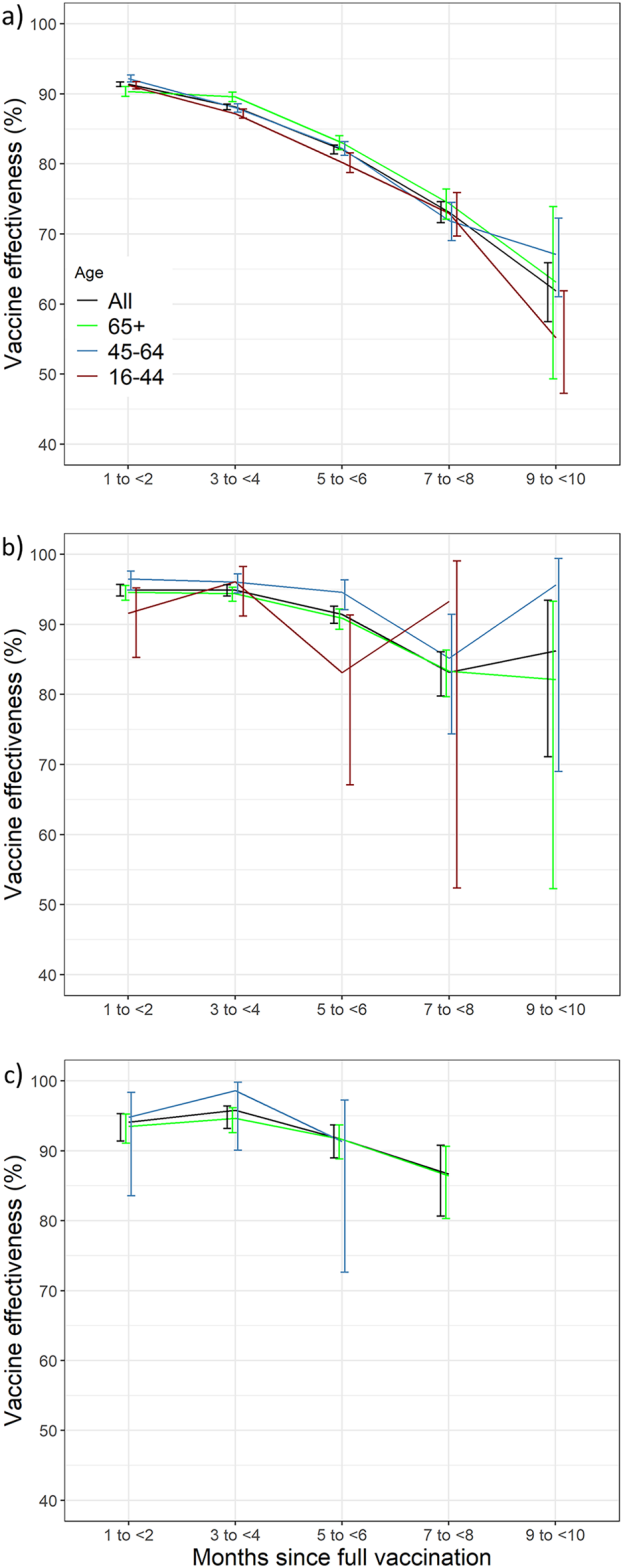
Adjusted vaccine effectiveness (estimates of COX models) of the vaccination programme against COVID-19 (**a**) infection, (**b**) hospitalisation, (**c**) death*, by age group and time since full vaccination. Apulia region, Italy, Jan 1–Dec, 2021. *Missing estimates due to limited follow-up time and/or events.

Stratified by time since vaccination, the effectiveness against infection for the total cohort gradually decreased from 91.37% (CI95% 87.73–88.18) during the 1–2 months after full vaccination to 88.14% (CI95% 87.76–88.52) after 3–4 months and to 61.91% (CI95% 57.47–65.89) after 9–10 months. Similar trends were observed for the various age categories (Fig. 4; SI Table 3a–d). The effectiveness against hospitalisation stratified by months since full vaccination for the total cohort showed a stable trend in the first six months (1–2 months: 94.94%, CI95% 94.04–95.65; 3–4 months: 94.92%, CI95% 94.06–95.65; 5–6 months: 91.45%, CI95% 90.13–92.59) after which it gradually reduced to 86.25% (CI95% 71.13–93.45) after nine to ten months (Fig. 4b; SI Table 3). The effectiveness against death stratified by months since full vaccination for the total cohort at 1–2 months and 7–8 months was 93.63% (CI95% 91.38–95.30) and 86.67% (CI95% 80.67–90.80, Fig. 4c; SI Table 3), respectively.

### Vaccination programme effectiveness by viral variants

**Figure 5:**
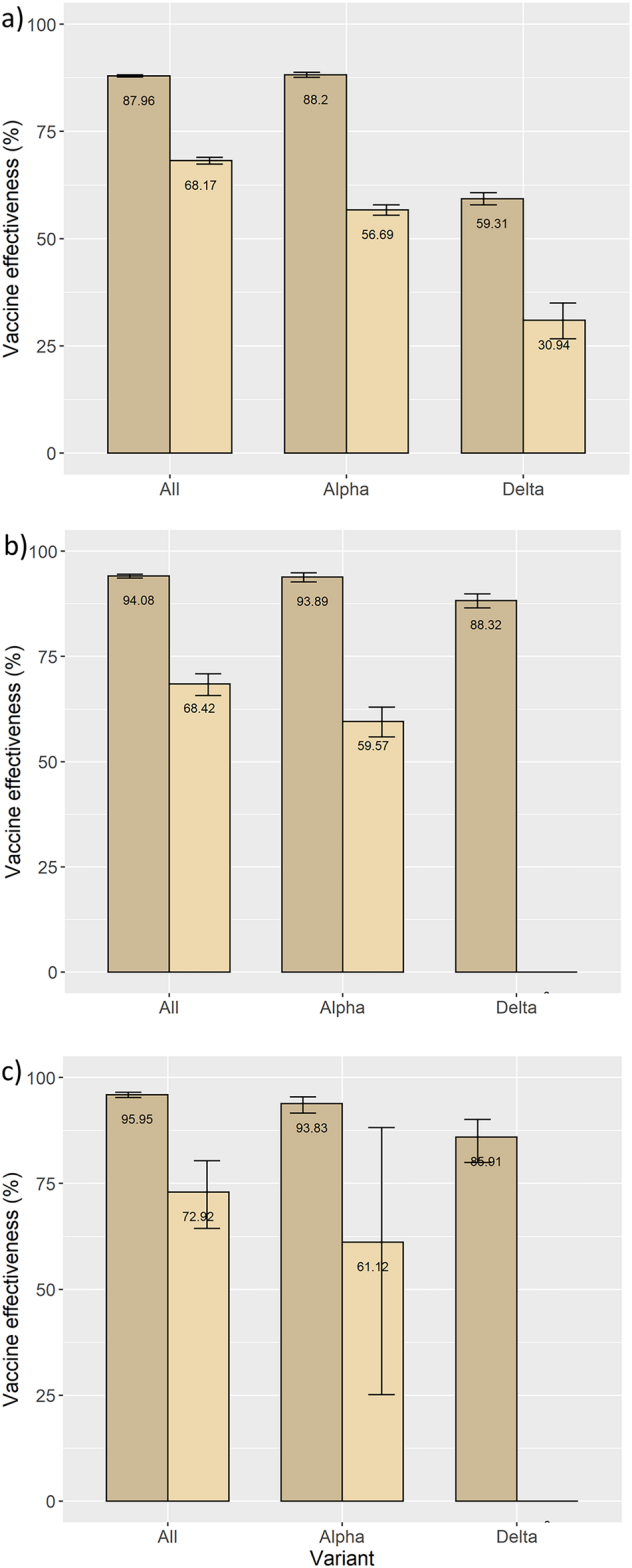
Adjusted vaccine effectiveness (estimates of COX models) of the vaccination programme against COVID-19 (**a**) infection, (**b**) hospitalisation, (**c**) death, by vaccination status and variant. Apulia region, Italy, Jan 1–Dec, 2021.

For the alpha variant, the adjusted effectiveness against infection was 88.20% (CI95% 87.60–88.78) for fully vaccinated and 56.69% (CI95% 55.50–57.86) for partially vaccinated individuals. The effectiveness against hospitalisation was 93.89% (CI95% 92.67–94.90) for fully and 59.57% (CI95% 55.88–62.94) for partially vaccinated persons. The effectiveness against death was 93.83% (CI95% 61.65–95.45) and 61.12% (CI95% 25.12–88.18) for fully and partially vaccinated individuals, respectively (Fig. 5; SI Table 4a).

For the delta variant, the adjusted effectiveness against infections was 59.31% (CI95% 57.91–60.67) for fully vaccinated and 30.94% (CI95% 26.62–35.01) for partially vaccinated persons. The effectiveness against hospitalisation was 88.32% (CI95% 86.50–89.90) and 85.91% (CI95% 79.98–90.09) against death (Fig. 5a,b,c; SI Table 4a,b).

**Figure 6:**
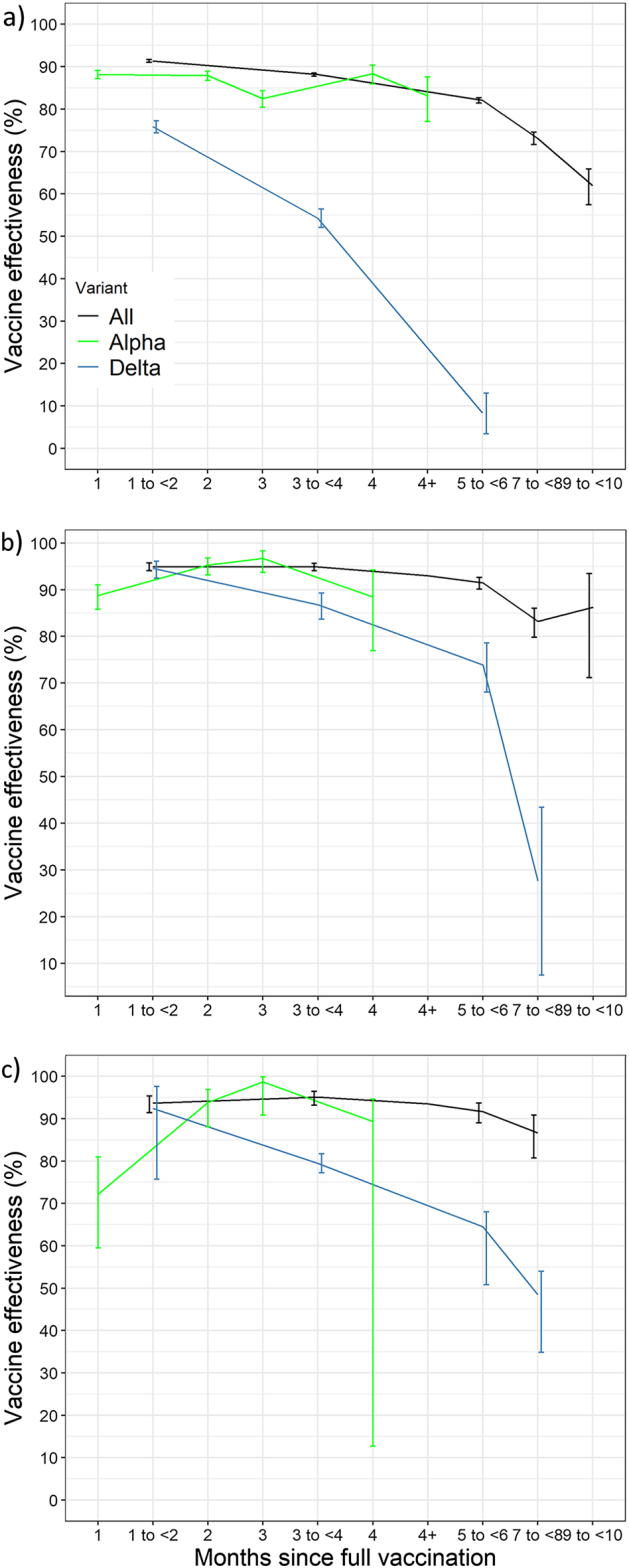
Adjusted vaccine effectiveness (estimates of COX models) of the vaccination programme against COVID-19 (**a**) infection, (**b**) hospitalisation, (**c**) death*, by variant** and time since full vaccination. Apulia region, Italy, Jan 1–Dec, 2021. *Missing estimates due to limited follow-up time and/or events. ** Estimates for alpha reported per single month given that maximum follow-up time was limited to 5.5 months.

Stratified by time since full vaccination, the effectiveness against infection with the alpha variant was 88.15% (CI95% 87.20–89.05) after one month after being fully vaccinated and 83.19% (CI95%77.09–87.54) after > 4 months while the effectiveness against infection with the delta variant was reduced from 75.85% (CI95% 74.38–77.24) during 1–2 months after full vaccination to 8.35% (CI95% 3.45–13.01) after 5–6 months and to − 55.99% (CI95% − 67.36 to − 45.39) after 7–8 months (Fig. 6a; SI Table 4a,b). The effectiveness against hospitalisation due to the alpha variant was 88.73% (CI95% 85.81–91.05) one month after being fully vaccinated and 88.46% (CI95%76.89–94.24) after > 4 months whereas the effectiveness against hospitalisation due to the delta variant was reduced from 94.57% (CI95% 92.45–96.10) at 1–2 months after full vaccination to 27.67% (CI95% 7.48–43.45) after 7–8 months (Fig. 6b; SI Table 4a,b). The effectiveness against death caused by the alpha variant was 93.83% (CI95% 88.04–96.82) two months after being fully vaccinated and 89.32% (CI95% 12.72–94.62) after 4 months whereas the effectiveness against death caused by the delta variant was reduced from 79.49% (CI95% 77.16–81.74) at 3–4 months after vaccination to 48.47% (CI95% 34.82–53.97) after 7–8 months (Fig. 6c; SI Table 4a,b).

The effectiveness against SARS-CoV-2 infections, COVID-19-related hospital admissions and deaths remain similar when we run the analysis separately for mRNA vaccines BNT162b2 and mRNA-1273 (SI, Tables 5a, 6a) and viral vector vaccines ChAdOx1-S and Ad26.COV2.S (SI, Tables 5b, 6b).

## Discussion

In our retrospective cohort study covering the first 11 months of 2021 in the Apulia region of Italy, we assessed the effectiveness of the combined anti-COVID-19 immunization programme including four vaccines: BNT162b2, mRNA-1273, ChAdOx1-S and Ad26.COV2.S. We estimate the vaccine effectiveness against SARS-CoV-2 infections, COVID-19-related hospital admissions and deaths. This study investigated the effectiveness of an entire vaccination programme, taking into account the waning effect of COVID-19 vaccines over time and the effect of different virus variants. We found that full vaccination was associated with large protection against SARS-CoV-2 related infection (87.96%, CI95% 87.73–88.18), hospitalisation (94.08, CI95% 93.58–94.54) and death (95.95%, CI95% 95.26–96.54). Moreover, the protective effect of full vaccination wanes gradually over time with the delta variant showing a more rapid decline.

Large cohort studies in European and America contexts report vaccine effectiveness estimations analogous to our study^23–25^. Minimal variations were observed among the different age groups. However, in line with findings elsewhere in Europe^24–26^, for those only partially vaccinated the effectiveness was lower, respectively with 68.17%, 68.42% and 72.92% with the 65 + year age group being considerably less protected (60.69%, 62.78% and 56.85%, Fig. 3).

The waning of effectiveness has been reported by numerous studies^13,18,19^, though the follow-up time was limited to 6 months, our study reports a period of 11 months. In a large cohort study in the USA investigating BNT162b2 effectiveness, the VE against infection and hospitalisation in a largely alpha variant dominated period respectively was reduced to around 50% and 85% for all age groups^13^. Whereas, in Israel the effectiveness against infection was still 84% four months after full vaccination^18^. In our study we found an effectiveness of 61.91% (CI95% 57.47–65.89) against infection only ten months after full vaccination, with 82.06% (CI95% 81.42–82.67) effectiveness after 5–6 months (Fig. 6b). Coherently, our finding on hospitalisation showed after six months an effectiveness of 91.45% (CI95% 90.13–92.59), reduced to 86.25% (CI95% 71.13–93.45) only after 9–10 months. The effectiveness against death remained high (91.66% after 5–6 months) similar to findings in Israel reporting 97% effectiveness over a six month period^10^, with only a prominent reduction after 7–8 months to 86.67% (CI95% 80.67–90.80) (Fig. 6c).

Our study covered a period in which at first the alpha variant was dominant followed in July by the delta variant being dominant in Italy^22^. By evaluating the first six months of 2021, we estimated the effectiveness against infection to be 88.20% (CI95% 87.60–88.78) for the alpha dominant period and 59.31% (CI95% 57.91–60.67) for the delta dominant period (July-November) (Fig. 4a). Therefore, the waning effect we observed over time might be due to the predominant circulation of the delta variant in the population. Our results are in line with other recent findings^23,27^ and meta-analyses^28,29^ confirming that full vaccination is associated with a wider reduced effectiveness against infection for the delta variant compared to that of the alpha variant. In one large cohort study^25^, effectiveness against infection for alpha and delta dominance was respectively 76% and 42% for BNT162b2 and 86% and 76% for mRNA-1273. In another US multi centre vaccination study effectiveness against alpha and delta variants was 91% and 66%, respectively^30^. Moreover, according to our findings, at 7–8 months after the second dose, effectiveness against delta variant infection was estimated to be similar to no protection. Analogously, drastic reduction in effectiveness against SARS-CoV-2 infection has been reported in Italy in high risk people (− 6%, CI95% − 28% to 12%) and in those aged ≥ 60 years (effectiveness 2%, CI95% − 11 to 14; 11%, CI95% − 15 to 31) at 27–30 weeks after the second dose^31^.

However, in our analysis, for hospitalisation due to COVID-19 over the study period the differences in effectiveness between alpha and delta remain modest (93.89% and 88.32%) consistent with a major study conducted in the UK^32^: effectiveness against hospitalisation for alpha and delta dominance was respectively 97% and 84% for BNT162b2 and 97% and 71% for ChAdOx1. Thus, the effectiveness against the delta variant is reduced considerably for infection, but to a lesser degree for COVID-19 attributed hospitalisation and death.

Although the gradual waning of vaccine effectiveness over time against various outcomes in the alpha dominant period is relatively well documented, this is not the case for the delta variant. We found that not only the effectiveness against infection with delta is much lower than with alpha, also the effectiveness wanes at a much higher pace with 75.85% effectiveness after 1–2 months and 8.35% after 5–6 months. In one study in the US the effectiveness of BNT162b2 against infection with delta was reduced to 61% after 3–4 months which in our study was 54.29%^13^. Moreover, we found that even the effectiveness against hospitalisation and death due to delta 1–2 months after full vaccination is reduced—the protection rapidly wanes indicating that after 7–8 month the effectiveness is respectively 27.67% and 48.47%.

Our study suggests that the estimates of effectiveness of an actual vaccine programme with multiple vaccines remains of high importance in controlling the COVID-19 pandemic. Full vaccination provides high levels of protection against hospitalisation and death for both variants of COVID-19. However, although the effectiveness against hospitalisation and death due to delta 1–2 months after full vaccination is reduced—the protection rapidly wanes indicating that after 7–8 months the effectiveness is respectively 27.67% and 48.47%. A systematic review of immunological studies on the waning effect after full vaccination with various vaccines find rapidly declining neutralising antibody titres patterns that are concordant with these findings^33^. Although the delta variant phase is now over, these results may be of interest to public health authorities, should a delta-like variant emerge.

### Limitations

Our study has several limitations to take into account. We estimated the effectiveness of the entire vaccination programme on COVID-19 outcomes, and we compared the undifferentiated results to those from results of analysis of effectiveness of mRNA and viral vector vaccines. Even though we do not report the vaccine effectiveness by brand, this approach does allow us to estimate a realistic real-world effect of the COVID-19 Vaccination programme, representative of those around Europe. This real-world example of an assessment of a combined vaccine effectiveness programme follows a field evaluation approach^34^ also deployed in various other studies^26,30,35^. Moreover, the distribution of fully vaccinated persons by type of vaccine in our study closely resemble those of major vaccine programmes in Europe as well the distribution of vaccines of all EU countries combined^36^.

Further, no individual data on virus strain was available for COVID-19 cases in the Apulia region for our analysis of the effectiveness against COVID-19 variants. To estimate the periods in which the alpha and delta variant were dominant, we consequently relied on a sample of sequenced results^22^. On basis of these data, we approximated the alpha (January–June) and delta (July–November) dominant period accepting that some of the COVID-19 cases in those periods were due to another variant. With the relatively more transmissible but less severe Omicron variant^37^ becoming dominant rapidly from December 2021 onwards^22^ as well as the booster dose becoming the rule rather than the exception, we attempted to reduce biases potentially arising from these dynamics by limiting the analysis until December 1.

Additionally, unlike randomised trials, our study design does not account for bias arising from variation in the study population. We considered for several factors that might have been unevenly distributed among the study population over time, including the calendar week (accounting for varying testing practices, progressive vaccine eligibility and various COVID-19 related restrictions) and week of infection (accounting for incidence of disease). Moreover, we were able to adjust for some critical personal factors that could potentially bias the effectiveness, including sex, age, residency, and fragility. However, the effectiveness estimates might be confounded due to skewed test-seeking behaviour among vaccinated and unvaccinated people and population groups with different socio-economic status^38^.

Moreover, including all individuals aged 65 + years as a single group might have masked differences in effectiveness between the younger and the oldest groups, among whom the risk of hospitalization and death was reported to be much higher^31^.

Finally, the interpretation of the results of the study compared to other vaccine programmes need to be done with some degree of precaution since every population in Europe has its own unique features making extrapolation a somewhat complicated.

## Conclusion

In conclusion, the vaccine programme in the Apulia region has showed to be very effective in averting deaths and hospitalisation against the alpha as well as delta variant, although its effectiveness is reduced for the latter. The waning of the effect after full vaccination against COVID-19 is evident, suggesting that after 4–6 months an extra dose is required. Encouragingly, recent studies on the effectiveness of a third dose have found that such boosters with BNT162b2 as well as with ChAdOx1 elicit even higher levels of serological responses than after two doses^39,40^. Booster doses of vaccines primarily to vulnerable people and to those 65 years and older is therefore of crucial importance in order to maintain adequate protection against hospitalisation and death by COVID-19 variants.

Additional studies to the effectiveness over time of the available vaccines against new variants such as Omicron are essential to further inform public health decision making.

## Supplementary Information

Supplementary Tables.

## Data Availability

The data that support the findings of this study are available from Public Health Authority of the Apulia Region but restrictions apply to the availability of these data, which were used under license for the current study, and so are not publicly available. Data are however available from the authors upon reasonable request and with permission of Apulian Public Health Authority.

## Abbreviations

Ad26.COV2.S: Janssen vaccine
AIFA: Agenzia italiana del farmaco
BNT162b2: Pfizer-BioNTech vaccine
ChAdOx1-S: Astra Zeneca vaccine
CI95%: 95% Confidence Interval
COVID-19: Coronavirus Disease 2019
HR: Hazard ratio
mRNA: Messenger Ribonucleic Acid
mRNA-1273: Moderna vaccine
NVX-CoV2373: Novavax vaccine
SARS-CoV-2: Severe acute respiratory syndrome coronavirus 2
SI: Supplementary Information
STROBE: Strengthening the Reporting of Observational Studies in Epidemiology
VE: Vaccine Effectiveness
