# Supplementary material for: Covid-19 vaccination programme effectiveness against SARS-CoV-2 related infections, hospital admissions and deaths in the Apulia region of Italy: a one-year retrospective cohort study
Source: Sci Rep. 2022 Nov 3;12:18597. doi: 10.1038/s41598-022-23235-4 (PMC9632568; doi:10.1038/s41598-022-23235-4)
Supplement: Supplementary file 1 — Supplementary Tables. [file 41598_2022_23235_MOESM1_ESM.pdf]

Supplementary Information

# Covid-19 vaccination programme effectiveness against SARS-CoV-2 related infections, hospital admissions and deaths in the Apulia region of Italy: a one-year retrospective cohort study

SI Table 1. COVID-19 infection, hospitalisation, death rates (per 100,000) due to all variants in all ages and per age category, by vaccination status and time since vaccination. Apulia region, Italy, Jan 1 – Dec 1, 2021

**a. All ages**

| Vaccination status | | P/years infection | P/years hospitalization | P/years death | Infection N. | Hospitalization N. | Deaths N. | Infection rate | Hospitalization rate | Death rate |
|---|---|---|---|---|---|---|---|---|---|---|
| Fully vaccinated | | 1,085,492.1 | 1,085,497.3 | 1,085,498.9 | 12,073 | 631 | 164 | 3.05 | 0.16 | 0.04 |
| | Time since vaccination, months | | | | | | | | | |
| | 1-2 | 395,572.3 | 395,577.6 | 395,574.1 | 2,923 | 147 | 44 | 2.02 | 0.10 | 0.03 |
| | 3-4 | 399,948.4 | 399,952.1 | 399,950.3 | 4,113 | 164 | 39 | 2.82 | 0.11 | 0.03 |
| | 5-6 | 223,915 | 223,917.5 | 223,916.6 | 3,419 | 197 | 52 | 4.18 | 0.24 | 0.06 |
| | 7-8 | 56,921.83 | 56,922.99 | 56,923.35 | 1,295 | 116 | 29 | 6.23 | 0.56 | 0.14 |
| | 9-10 | 9,073.682 | 9,073.762 | 9,073.682 | 319 | 7 | 0 | 9.63 | 0.21 | 0.00 |
| Partially vaccinated | | 259,852.9 | 259,865.6 | 259,869.5 | 7,799 | 659 | 236 | 8.21 | 0.69 | 0.25 |
| Unvaccinated | | 1,685,329.2 | 1,685,479.3 | 1,685,497.9 | 138,441 | 11,253 | 3,258 | 22.50 | 1.83 | 0.53 |
| Total | | 3,030,674 | 3,030,842.2 | 3,030,866.3 | 158,313 | 12,543 | 3,658 | 5.22 | 0.41 | 0.12 |

**b. Persons 65+ years old**

| Vaccination status | | P/years infection | P/years hospitalization | P/years death | Infection N. | Hospitalization N. | Deaths N. | Infection rate | Hospitalization rate | Death rate |
|---|---|---|---|---|---|---|---|---|---|---|
| Fully vaccinated | | 378,388.36 | 378,392.56 | 378,394.96 | 3,644 | 496 | 157 | 2.64 | 0.36 | 0.11 |
| | Time since vaccination, months | | | | | | | | | |
| | 1-2 | 117,256.1 | 117,260.3 | 117,257.8 | 813 | 106 | 41 | 1.90 | 0.25 | 0.10 |
| | 3-4 | 130,038.8 | 130,041.8 | 130,040.6 | 980 | 123 | 38 | 2.06 | 0.26 | 0.08 |
| | 5-6 | 99,749.85 | 99,751.88 | 99,751.41 | 1,233 | 161 | 49 | 3.39 | 0.44 | 0.13 |
| | 7-8 | 29,993.87 | 29,994.92 | 29,995.39 | 580 | 102 | 29 | 5.30 | 0.93 | 0.26 |
| | 9-10 | 1,346.162 | 1,346.222 | 1,346.162 | 38 | 4 | 0 | 7.73 | 0.81 | 0.00 |
| Partially vaccinated | | 83,726.39 | 83,736.89 | 83,742.45 | 2,656 | 520 | 227 | 8.69 | 1.70 | 0.74 |
| Unvaccinated | | 354,745.87 | 354,826.02 | 354,890.96 | 27,562 | 6,568 | 2,806 | 21.29 | 5.07 | 2.17 |

**c. Persons 45-64 years old**

| Vaccination status | | P/years infection | P/years hospitalization | P/years death | Infection N. | Hospitalization N. | Deaths N. | Infection rate | Hospitalization rate | Death rate |
|---|---|---|---|---|---|---|---|---|---|---|
| Fully vaccinated | | 391,401.34 | 391,402.12 | 391,401.52 | 4,528 | 104 | 7 | 3.17 | 0.07 | 0.00 |
| | Time since vaccination, months | | | | | | | | | |
| | 1-2 | 139,786.9 | 139,786.2 | 971 | 28 | 3 | 1.90 | 0.05 | 0.01 | 139,786.9 |
| | 3-4 | 147,493.5 | 147,492.9 | 1,617 | 35 | 1 | 3.00 | 0.07 | 0.0 | 147,493.5 |
| | 5-6 | 83,800.46 | 83,800.18 | 1,386 | 27 | 3 | 4.53 | 0.09 | 0.01 | 83,800.46 |
| | 7-8 | 15,864.84 | 15,864.76 | 418 | 13 | 0 | 7.22 | 0.22 | 0.0 | 15,864.84 |
| | 9-10 | 4,428.748 | 4,428.734 | 134 | 1 | 0 | 8.29 | 0.06 | 0.0 | 4,428.748 |

| | | | | | | | | | |
|---|---|---|---|---|---|---|---|---|---|
| Partially vaccinated | 93,897.15 | 93,895.93 | 2,609 | 113 | 9 | 7.61 | 0.33 | 0.03 | 93,897.15 |
| Unvaccinated | 573,822.82 | 573,800.49 | 49,408 | 3,662 | 426 | 23.59 | 1.75 | 0.20 | 573,822.82 |

**d. Persons 16-44 years old**

| Vaccination status | P/years infection | P/years hospitalization | P/years death | Infection N. | Hospitalization N. | Deaths N. | Infection rate | Hospitalisation rate | Death rate |
|---|---|---|---|---|---|---|---|---|---|
| Fully vaccinated | 315,702.38 | 315,702.62 | 315,702.38 | 3,898 | 31 | 0 | 3.38 | 0.03 | 0.00 |
| Time since vaccination, months | | | | | | | | | |
| 1-2 | 138,530.1 | 138,530.4 | 138,530.1 | 1,137 | 13 | 0 | 2.25 | 0.03 | 0.00 |
| 3-4 | 122,416.7 | 122,416.8 | 122,416.7 | 1,515 | 6 | 0 | 3.39 | 0.01 | 0.00 |
| 5-6 | 40,365.03 | 40,365.15 | 40,365.03 | 800 | 9 | 0 | 5.43 | 0.06 | 0.00 |
| 7-8 | 11,063.21 | 11,063.23 | 11,063.21 | 297 | 1 | 0 | 7.35 | 0.02 | 0.00 |
| 9-10 | 3,298.786 | 3,298.792 | 3,298.786 | 147 | 2 | 0 | 12.21 | 0.17 | 0.00 |
| Partially | 82,231.15 | 82,231.58 | 82,231.15 | 2,521 | 26 | 0 | 8.40 | 0.09 | 0.00 |
| Unvaccinated | 756,805.19 | 756,830.48 | 756,806.42 | 61,458 | 1,023 | 26 | 22.25 | 0.37 | 0.01 |

SI Table 2. COVID-19 infection, hospitalisation, death rates (per 100,000) in all ages, per variant, by vaccination status and time since vaccination. Apulia region, Italy, Jan 1 – Dec 1, 2021

**a. alpha variant**

| Vaccination status | **P/years infection** | **P/years hospitalization** | **P/years death** | **Infection N.** | **Hospitalization N.** | **Deaths N.** | **Infection rate** | **Hospitalization rate** | **Death rate** |
|---|---|---|---|---|---|---|---|---|---|
| Fully vaccinated | 143,477.0 | 143,481.6 | 143,478.8 | 1,589 | 120 | 43 | 3.03 | 0.23 | 0.08 |
| Time since vaccination, months | | | | | | | | | |
| 1 | 61,426.5 | 61,431.15 | 61,427.64 | 654 | 76 | 31 | 2.92 | 0.34 | 0.14 |
| 2 | 45,983.91 | 45,987.66 | 45,984.42 | 489 | 27 | 9 | 2.91 | 0.16 | 0.05 |
| 3 | 20,872.81 | 20,874.56 | 20,872.82 | 320 | 9 | 1 | 4.2 | 0.12 | 0.01 |
| 4 | 9,683.545 | 9,684.595 | 9,683.715 | 106 | 8 | 2 | 3.0 | 0.23 | 0.06 |
| >4 | 5,510.2 | 5,510.789 | 5,510.2 | 20 | 0 | 0 | 0.99 | 0.00 | 0.00 |
| Partially vaccinated | 153,451.1 | 153,463.7 | 153,462.7 | 6,107 | 608 | 232 | 10.90 | 1.09 | 0.41 |
| Unvaccinated | 1,381,248.5 | 1,381,398.6 | 1,381,408.5 | 131,585 | 10,689 | 3,188 | 26.10 | 2.12 | 0.63 |

**b. delta variant**

| Vaccination status | **P/years infection** | **P/years hospitalization** | **P/years death** | **Infection N.** | **Hospitalization N.** | **Deaths N.** | **Infection rate** | **Hospitalization rate** | **Death rate** |
|---|---|---|---|---|---|---|---|---|---|
| Fully vaccinated | 1,085,068.9 | 1,085,072.8 | 1,085,073.9 | 10,484 | 512 | 121 | 2.65 | 0.13 | 0.03 |
| Time since vaccination, months | | | | | | | | | |
| 1-2 | 349,071.6 | 349,073.8 | 349,071.7 | 1,779 | 44 | 4 | 1.40 | 0.03 | 0.00 |
| 3-4 | 387,952.0 | 387,955 | 387,953.6 | 3,688 | 148 | 36 | 2.60 | 0.1 | 0.03 |
| 5-6 | 223,909.4 | 223,911.9 | 223,911.0 | 3,399 | 197 | 52 | 4.16 | 0.24 | 0.06 |
| 7-8 | 56,921.83 | 56,922.99 | 56,923.35 | 1,295 | 116 | 29 | 6.23 | 0.56 | 0.14 |
| 9-10 | 9,073.682 | 9,073.762 | 9,073.682 | 319 | 7 | 0 | 9.63 | 0.21 | 0.00 |
| Partially vaccinated | 168,633.2 | 168,633.9 | 168,633.6 | 1,692 | 53 | 4 | 2.75 | 0.09 | 0.01 |
| Unvaccinated | 307,457.8 | 307,463.0 | 307,461.5 | 6,856 | 564 | 70 | 6.11 | 0.50 | 0.06 |

SI Table 3a: Covid-19 infection, hospitalization, and death hazard ratios (HR) per 100.000 person days and adjusted VE estimates using Cox regression in **all ages for all variants**, by vaccination status and time since vaccination. Apulia region, Italy, Jan 1 – Dec, 2021

| **Infection**<br>Vaccination status | **HR** | **HR 95% CI**<br>**low** | **up** | **Adj IRR** | **Adj HR 95% CI**<br>**low** | **up** | **Adj VE** | **Adj VE 95% CI**<br>**low** | **up** |
|---|---|---|---|---|---|---|---|---|---|
| Fully vaccinated | 0.119 | 0.117 | 0.121 | 0.1204 | 0.1182 | 0.1227 | 87.96 | 87.73 | 88.18 |
| Time since vaccination, months | | | | | | | | | |
| 1-2 | 0.086 | 0.083 | 0.089 | 0.0863 | 0.0831 | 0.0896 | 91.37 | 91.04 | 91.69 |
| 3-4 | 0.118 | 0.114 | 0.121 | 0.1186 | 0.1148 | 0.1224 | 88.14 | 87.76 | 88.52 |
| 5-6 | 0.173 | 0.167 | 0.179 | 0.1794 | 0.1733 | 0.1858 | 82.06 | 81.42 | 82.67 |
| 7-8 | 0.252 | 0.238 | 0.266 | 0.2687 | 0.2541 | 0.2841 | 73.13 | 71.59 | 74.59 |
| 9-10 | 0.390 | 0.349 | 0.435 | 0.3809 | 0.3411 | 0.4253 | 61.91 | 57.47 | 65.89 |
| Partially | 0.313 | 0.305 | 0.320 | 0.3183 | 0.3107 | 0.3260 | 68.17 | 67.40 | 68.93 |

| **Hospitalization**<br>Vaccination status | **HR** | **HR 95% CI**<br>**low** | **up** | **Adj IRR** | **Adj HR 95% CI**<br>**low** | **up** | **Adj VE** | **Adj VE 95% CI**<br>**low** | **up** |
|---|---|---|---|---|---|---|---|---|---|
| Fully vaccinated | 0.077 | 0.071 | 0.083 | 0.0592 | 0.0546 | 0.0642 | 94.08 | 93.58 | 94.54 |
| Time since vaccination, months | | | | | | | | | |
| 1-2 | 0.053 | 0.045 | 0.063 | 0.0506 | 0.0429 | 0.0596 | 94.94 | 94.04 | 95.71 |
| 3-4 | 0.058 | 0.049 | 0.068 | 0.0508 | 0.0435 | 0.0594 | 94.92 | 94.06 | 95.65 |
| 5-6 | 0.127 | 0.110 | 0.147 | 0.0855 | 0.0741 | 0.0987 | 91.45 | 90.13 | 92.59 |
| 7-8 | 0.301 | 0.250 | 0.362 | 0.1680 | 0.1394 | 0.2023 | 83.20 | 79.77 | 86.06 |
| 9-10 | 0.115 | 0.055 | 0.241 | 0.1375 | 0.0655 | 0.2887 | 86.25 | 71.13 | 93.45 |
| Partially vaccinated | 0.345 | 0.317 | 0.374 | 0.3158 | 0.2909 | 0.3429 | 68.42 | 65.71 | 70.91 |

| **Death**<br>Vaccination status | **HR** | **HR 95% CI**<br>**low** | **up** | **Adj IRR** | **Adj HR 95% CI**<br>**low** | **up** | **Adj VE** | **Adj VE 95% CI**<br>**low** | **up** |
|---|---|---|---|---|---|---|---|---|---|
| Fully vaccinated | 0.068 | 0.058 | 0.079 | 0.0405 | 0.0346 | 0.0474 | 95.95 | 95.26 | 96.54 |
| Time since vaccination, months | | | | | | | | | |
| 1-2 | 0.067 | 0.049 | 0.090 | 0.0637 | 0.0470 | 0.0862 | 93.63 | 91.38 | 95.30 |
| 3-4 | 0.058 | 0.042 | 0.079 | 0.0494 | 0.0359 | 0.0681 | 95.06 | 93.19 | 96.41 |
| 5-6 | 0.150 | 0.114 | 0.199 | 0.0834 | 0.0631 | 0.1102 | 91.66 | 88.98 | 93.69 |
| 7-8 | 0.372 | 0.257 | 0.539 | 0.1333 | 0.0920 | 0.1933 | 86.67 | 80.67 | 90.80 |
| 9-10 | - | - | - | - | - | - | - | - | - |
| Partially vaccinated | 0.599 | 0.521 | 0.690 | 0.2708 | 0.1965 | 0.3561 | 72.92 | 64.39 | 80.35 |

SI Table 3b: Covid-19 infection, hospitalization, and death hazard ratios (HR) per 100.000 person days and the VE estimates using Cox regression in **65+ years old persons and all variants**, by vaccination status and time since vaccination. Apulia region, Italy, Jan 1 – Dec, 2021

| **Infection**<br>Vaccination status | **HR** | **HR 95% CI**<br>**low** | **up** | **Adj IRR** | **Adj HR 95% CI**<br>**low** | **up** | **Adj VE** | **Adj VE 95% CI**<br>**low** | **up** |
|---|---|---|---|---|---|---|---|---|---|
| Fully vaccinated | 0.125 | 0.121 | 0.130 | 0.1238 | 0.1195 | 0.1282 | 87.62 | 87.18 | 88.05 |
| Time since vaccination, months | | | | | | | | | |
| 1-2 | 0.097 | 0.090 | 0.104 | 0.0965 | 0.0898 | 0.1036 | 90.35 | 89.64 | 91.02 |
| 3-4 | 0.104 | 0.098 | 0.111 | 0.1041 | 0.0976 | 0.1112 | 89.59 | 88.88 | 90.24 |
| 5-6 | 0.170 | 0.160 | 0.180 | 0.1696 | 0.1599 | 0.1799 | 83.04 | 82.01 | 84.01 |
| 7-8 | 0.258 | 0.237 | 0.280 | 0.2565 | 0.2356 | 0.2791 | 74.35 | 72.09 | 76.44 |
| 9-10 | 0.371 | 0.269 | 0.510 | 0.3685 | 0.2678 | 0.5071 | 63.15 | 49.29 | 73.92 |
| Partially | 0.388 | 0.372 | 0.404 | 0.3931 | 0.3770 | 0.4099 | 60.69 | 59.01 | 62.30 |

| **Hospitalization**<br>Vaccination status | **HR** | **HR 95% CI**<br>**low** | **up** | **Adj IRR** | **Adj HR 95% CI**<br>**low** | **up** | **Adj VE** | **Adj VE 95% CI**<br>**low** | **up** |
|---|---|---|---|---|---|---|---|---|---|
| Fully vaccinated | 0.072 | 0.066 | 0.079 | 0.0672 | 0.0613 | 0.0737 | 93.28 | 92.63 | 93.87 |
| Time since vaccination, months | | | | | | | | | |
| 1-2 | 0.054 | 0.044 | 0.066 | 0.0541 | 0.0445 | 0.0657 | 94.59 | 93.43 | 95.55 |
| 3-4 | 0.056 | 0.047 | 0.067 | 0.0560 | 0.0468 | 0.0672 | 94.40 | 93.28 | 95.32 |
| 5-6 | 0.096 | 0.082 | 0.113 | 0.0912 | 0.0778 | 0.1070 | 90.88 | 89.30 | 92.22 |
| 7-8 | 0.208 | 0.170 | 0.253 | 0.1666 | 0.1364 | 0.2034 | 83.34 | 79.66 | 86.36 |
| 9-10 | 0.1940 | 0.073 | 0.519 | 0.1787 | 0.0669 | 0.4771 | 82.13 | 52.29 | 93.31 |
| Partially vaccinated | 0.342 | 0.311 | 0.375 | 0.3722 | 0.3388 | 0.4087 | 62.78 | 59.13 | 66.12 |

| **Death**<br>Vaccination status | **HR** | **HR 95% CI**<br>**low** | **up** | **Adj IRR** | **Adj HR 95% CI**<br>**low** | **up** | **Adj VE** | **Adj VE 95% CI**<br>**low** | **up** |
|---|---|---|---|---|---|---|---|---|---|
| Fully vaccinated | 0.048 | 0.041 | 0.056 | 0.0421 | 0.0358 | 0.0495 | 95.79 | 95.05 | 96.42 |
| Time since vaccination, months | | | | | | | | | |
| 1-2 | 0.063 | 0.046 | 0.086 | 0.0652 | 0.0476 | 0.0893 | 93.48 | 91.07 | 95.24 |
| 3-4 | 0.052 | 0.038 | 0.072 | 0.0535 | 0.0386 | 0.0740 | 94.65 | 92.60 | 96.14 |
| 5-6 | 0.092 | 0.069 | 0.122 | 0.0838 | 0.0629 | 0.1118 | 91.62 | 88.82 | 93.71 |
| 7-8 | 0.221 | 0.153 | 0.321 | 0.1358 | 0.0936 | 0.1971 | 86.42 | 80.29 | 90.64 |
| 9-10 | - | - | - | - | - | - | - | - | - |
| Partially vaccinated | 0.491 | 0.426 | 0.565 | 0.4320 | 0.3476 | 0.5293 | 56.80 | 47.07 | 65.24 |

SI Table 3c: Covid-19 infection, hospitalization, and death hazard ratios (HR) per 100.000 person days and the VE estimates using Cox regression in **44-65 years old persons for all variants**, by vaccination status and time since vaccination. Apulia region, Italy, Jan 1 – Dec, 2021

| **Infection**<br>Vaccination status | **HR** | **HR 95% CI**<br>**low** | <br>**up** | **Adj IRR** | **Adj HR 95% CI**<br>**low** | <br>**up** | **Adj VE** | **Adj VE 95% CI**<br>**low** | <br>**up** |
|---|---|---|---|---|---|---|---|---|---|
| Fully vaccinated | 0.118 | 0.115 | 0.122 | 0.1172 | 0.1137 | 0.1208 | 88.28 | 87.92 | 88.63 |
| Time since vaccination, months | | | | | | | | | |
| 1-2 | 0.078 | 0.073 | 0.083 | 0.0778 | 0.0729 | 0.0830 | 92.22 | 91.70 | 92.71 |
| 3-4 | 0.121 | 0.115 | 0.127 | 0.1198 | 0.1139 | 0.1261 | 88.02 | 87.39 | 88.61 |
| 5-6 | 0.181 | 0.171 | 0.191 | 0.1775 | 0.1680 | 0.1875 | 82.25 | 81.25 | 83.20 |
| 7-8 | 0.283 | 0.257 | 0.312 | 0.2807 | 0.2547 | 0.3094 | 71.93 | 69.06 | 74.53 |
| 9-10 | 0.328 | 0.277 | 0.389 | 0.3288 | 0.2774 | 0.3898 | 67.12 | 61.02 | 72.26 |
| Partially | 0.287 | 0.275 | 0.299 | 0.2871 | 0.2755 | 0.2991 | 71.29 | 70.09 | 72.45 |

| **Hospitalization**<br>Vaccination status | **HR** | **HR 95% CI**<br>**low** | <br>**up** | **Adj IRR** | **Adj HR 95% CI**<br>**low** | <br>**up** | **Adj VE** | **Adj VE 95% CI**<br>**low** | <br>**up** |
|---|---|---|---|---|---|---|---|---|---|
| Fully vaccinated | 0.037 | 0.031 | 0.045 | 0.0361 | 0.0297 | 0.0439 | 96.39 | 95.61 | 97.03 |
| Time since vaccination, months | | | | | | | | | |
| 1-2 | 0.035 | 0.024 | 0.052 | 0.0350 | 0.0240 | 0.0509 | 96.50 | 94.91 | 97.60 |
| 3-4 | 0.040 | 0.029 | 0.056 | 0.0394 | 0.0281 | 0.0551 | 96.06 | 94.49 | 97.19 |
| 5-6 | 0.058 | 0.040 | 0.086 | 0.0538 | 0.0367 | 0.0789 | 94.62 | 92.11 | 96.33 |
| 7-8 | 0.151 | 0.087 | 0.261 | 0.1483 | 0.0858 | 0.2564 | 85.17 | 74.36 | 91.42 |
| 9-10 | 0.042 | 0.006 | 0.300 | 0.0436 | 0.0061 | 0.3098 | 95.64 | 69.02 | 99.39 |
| Partially vaccinated | 0.209 | 0.172 | 0.254 | 0.1979 | 0.1632 | 0.2400 | 80.21 | 76.00 | 83.68 |

| **Death**<br>Vaccination status | **HR** | **HR 95% CI**<br>**low** | <br>**up** | **Adj IRR** | **Adj HR 95% CI**<br>**low** | <br>**up** | **Adj VE** | **Adj VE 95% CI**<br>**low** | <br>**up** |
|---|---|---|---|---|---|---|---|---|---|
| Fully vaccinated | 0.021 | 0.010 | 0.045 | 0.0203 | 0.0096 | 0.0429 | 97.97 | 95.71 | 99.04 |
| Time since vaccination, months | | | | | | | | | |
| 1-2 | 0.053 | 0.017 | 0.167 | 0.0519 | 0.0163 | 0.1646 | 94.81 | 83.54 | 98.37 |
| 3-4 | 0.014 | 0.002 | 0.102 | 0.0138 | 0.0019 | 0.0993 | 98.62 | 90.07 | 99.81 |
| 5-6 | 0.094 | 0.030 | 0.296 | 0.0865 | 0.0273 | 0.2735 | 91.35 | 72.65 | 97.27 |
| 7-8 | - | - | - | - | - | - | - | - | - |
| 9-10 | - | - | - | - | - | - | - | - | - |
| Partially vaccinated | 0.225 | 0.114 | 0.442 | 0.1810 | 0.0923 | 0.3550 | 81.90 | 64.50 | 90.77 |

SI Table 3d: Covid-19 infection, hospitalization, and death hazard ratios (HR) per 100.000 person days and the VE estimates using Cox regression in **16-44 years old persons for all variants**, by vaccination status and time since vaccination. Apulia region, Italy, Jan 1 – Dec, 2021

| **Infection** | **HR** | **HR 95% CI** | | **Adj IRR** | **Adj HR 95% CI** | | **Adj VE** | **Adj VE 95% CI** | |
|---|---|---|---|---|---|---|---|---|---|
| Vaccination status | | **low** | **up** | | **low** | **up** | | **low** | **up** |
| Fully vaccinated | 0.116 | 0.112 | 0.120 | 0.1142 | 0.1106 | 0.1180 | 88.58 | 88.20 | 88.94 |
| Time since vaccination, months | | | | | | | | | |
| 1-2 | 0.088 | 0.083 | 0.094 | 0.0876 | 0.0825 | 0.0930 | 91.24 | 90.70 | 91.75 |
| 3-4 | 0.130 | 0.124 | 0.137 | 0.1280 | 0.1215 | 0.1348 | 87.20 | 86.52 | 87.85 |
| 5-6 | 0.202 | 0.188 | 0.217 | 0.1977 | 0.1842 | 0.2123 | 80.23 | 78.77 | 81.58 |
| 7-8 | 0.275 | 0.245 | 0.308 | 0.2702 | 0.2409 | 0.3030 | 72.98 | 69.70 | 75.91 |
| 9-10 | 0.453 | 0.385 | 0.532 | 0.4484 | 0.3811 | 0.5274 | 55.16 | 47.26 | 61.89 |
| Partially | 0.296 | 0.284 | 0.309 | 0.2947 | 0.2825 | 0.3073 | 70.53 | 69.27 | 71.75 |

| **Hospitalization** | **HR** | **HR 95% CI** | | **Adj IRR** | **Adj HR 95% CI** | | **Adj VE** | **Adj VE 95% CI** | |
|---|---|---|---|---|---|---|---|---|---|
| Vaccination status | | **low** | **up** | | **low** | **up** | | **low** | **up** |
| Fully vaccinated | 0.063 | 0.044 | 0.091 | 0.0608 | 0.0425 | 0.0870 | 93.92 | 91.30 | 95.75 |
| Time since vaccination, months | | | | | | | | | |
| 1-2 | 0.083 | 0.047 | 0.145 | 0.0841 | 0.0481 | 0.1471 | 91.59 | 85.29 | 95.19 |
| 3-4 | 0.041 | 0.018 | 0.092 | 0.0392 | 0.0175 | 0.0881 | 96.08 | 91.19 | 98.25 |
| 5-6 | 0.197 | 0.101 | 0.383 | 0.1689 | 0.0868 | 0.3288 | 83.11 | 67.12 | 91.32 |
| 7-8 | 0.077 | 0.011 | 0.550 | 0.0668 | 0.0094 | 0.4763 | 93.32 | 52.37 | 99.06 |
| 9-10 | 0.548 | 0.136 | 2.207 | 0.4612 | 0.1145 | 1.8570 | 53.88 | -85.70 | 88.55 |
| Partially vaccinated | 0.262 | 0.174 | 0.394 | 0.2601 | 0.1730 | 0.3910 | 73.99 | 60.90 | 82.70 |

| **Death** | **HR** | **HR 95% CI** | | **Adj IRR** | **Adj HR 95% CI** | | **Adj VE** | **Adj VE 95% CI** | |
|---|---|---|---|---|---|---|---|---|---|
| Vaccination status | | **low** | **up** | | **low** | **up** | | **low** | **up** |
| Fully vaccinated | - | - | - | - | - | - | - | - | - |
| Time since vaccination, months | | | | | | | | | |
| 1-2 | - | - | - | - | - | - | - | - | - |
| 3-4 | - | - | - | - | - | - | - | - | - |
| 5-6 | - | - | - | - | - | - | - | - | - |
| 7-8 | - | - | - | - | - | - | - | - | - |
| 9-10 | - | - | - | - | - | - | - | - | - |
| Partially vaccinated | - | - | - | - | - | - | - | - | - |

SI Table 4a: Covid-19 infection, hospitalization, and death hazard ratios (HR) per 100.000 person days and the VE estimates using Cox regression in **all ages for alpha variant**, by vaccination status and time since vaccination. Apulia region, Italy, Jan 1 – Dec, 2021

| **Infection**<br>Vaccination status | | **HR** | **HR 95% CI** | | **Adj IRR** | **Adj HR 95% CI** | | **Adj VE** | **Adj VE 95% CI** | |
|---|---|---|---|---|---|---|---|---|---|---|
| | | | **low** | **up** | | **low** | **up** | | **low** | **up** |
| Fully vaccinated | | 0.112 | 0.107 | 0.118 | 0.1180 | 0.1122 | 0.1240 | 88.20 | 87.60 | 88.78 |
| Time since vaccination, months | | | | | | | | | | |
| | 1 | 0.113 | 0.104 | 0.122 | 0.1184 | 0.1095 | 0.1280 | 88.16 | 87.20 | 89.05 |
| | 2 | 0.114 | 0.104 | 0.124 | 0.1212 | 0.1108 | 0.1326 | 87.88 | 86.74 | 88.92 |
| | 3 | 0.166 | 0.148 | 0.185 | 0.1754 | 0.1570 | 0.1959 | 82.46 | 80.41 | 84.30 |
| | 4 | 0.116 | 0.096 | 0.141 | 0.1165 | 0.0963 | 0.1410 | 88.35 | 85.90 | 90.37 |
| | >4 | 0.039 | 0.025 | 0.061 | 0.1681 | 0.0246 | 0.1291 | 83.19 | 77.09 | 87.54 |
| Partially | | 0.419 | 0.408 | 0.430 | 0.4331 | 0.4214 | 0.4450 | 56.69 | 55.50 | 57.86 |

| **Hospitalization**<br>Vaccination status | | **HR** | **HR 95% CI** | | **Adj IRR** | **Adj HR 95% CI** | | **Adj VE** | **Adj VE 95% CI** | |
|---|---|---|---|---|---|---|---|---|---|---|
| | | | **low** | **up** | | **low** | **up** | | **low** | **up** |
| Fully vaccinated | | 0.108 | 0.090 | 0.129 | 0.0611 | 0.0510 | 0.0733 | 93.89 | 92.67 | 94.90 |
| Time since vaccination, months | | | | | | | | | | |
| | 1 | 0.188 | 0.150 | 0.237 | 0.1127 | 0.0895 | 0.1419 | 88.73 | 85.81 | 91.05 |
| | 2 | 0.089 | 0.061 | 0.131 | 0.0469 | 0.0321 | 0.0686 | 95.31 | 93.14 | 96.79 |
| | 3 | 0.065 | 0.034 | 0.125 | 0.0329 | 0.0171 | 0.0633 | 96.71 | 93.67 | 98.29 |
| | 4 | 0.128 | 0.064 | 0.256 | 0.1154 | 0.0576 | 0.2311 | 88.46 | 76.89 | 94.24 |
| | >4 | - | - | - | - | - | - | - | - | - |
| Partially vaccinated | | 0.551 | 0.505 | 0.601 | 0.4043 | 0.3706 | 0.4412 | 59.57 | 55.88 | 62.94 |

| **Death**<br>Vaccination status | | **HR** | **HR 95% CI** | | **Adj IRR** | **Adj HR 95% CI** | | **Adj VE** | **Adj VE 95% CI** | |
|---|---|---|---|---|---|---|---|---|---|---|
| | | | **low** | **up** | | **low** | **up** | | **low** | **up** |
| Fully vaccinated | | 0.157 | 0.116 | 0.212 | 0.0617 | 0.0455 | 0.0835 | 93.83 | 91.65 | 95.45 |
| Time since vaccination, months | | | | | | | | | | |
| | 1 | 0.585 | 0.401 | 0.853 | 0.2781 | 0.1907 | 0.4055 | 72.19 | 59.45 | 80.93 |
| | 2 | 0.182 | 0.094 | 0.353 | 0.0617 | 0.0318 | 0.1196 | 93.83 | 88.04 | 96.82 |
| | 3 | 0.041 | 0.006 | 0.290 | 0.0129 | 0.0018 | 0.0921 | 98.71 | 90.79 | 99.82 |
| | 4 | 0.298 | 0.149 | 0.896 | 0.1068 | 0.0538 | 0.8727 | 89.32 | 12.73 | 94.62 |
| | >4 | - | - | - | - | - | - | - | - | - |
| Partially vaccinated | | 0.5624 | 0.1295 | 0.8515 | 0.3888 | 0.1182 | 0.7488 | 61.12 | 25.12 | 88.18 |

SI Table 4b: Covid-19 infection, hospitalization, and death hazard ratios (HR) per 100.000 person days and the VE estimates using Cox regression in **all ages for delta variants**, by vaccination status and time since vaccination. Apulia region, Italy, Jan 1 – Dec, 2021

| **Infection** | **HR** | **HR 95% CI** | | **Adj IRR** | **Adj HR 95% CI** | | **Adj VE** | **Adj VE 95% CI** | |
|---|---|---|---|---|---|---|---|---|---|
| Vaccination status | | **low** | **up** | | **low** | **up** | | **low** | **up** |
| Fully vaccinated | 0.355 | 0.344 | 0.367 | 0.4069 | 0.3933 | 0.4209 | 59.31 | 57.91 | 60.67 |
| Time since vaccination, months | | | | | | | | | |
| 1-2 | 0.210 | 0.199 | 0.223 | 0.2415 | 0.2276 | 0.2562 | 75.85 | 74.38 | 77.24 |
| 3-4 | 0.378 | 0.361 | 0.395 | 0.4571 | 0.4358 | 0.4794 | 54.29 | 52.06 | 56.42 |
| 5-6 | 0.661 | 0.631 | 0.693 | 0.9165 | 0.8699 | 0.9655 | 8.35 | 3.45 | 13.01 |
| 7-8 | 1.119 | 1.050 | 1.193 | 1.5599 | 1.4539 | 1.6736 | -55.99 | -67.36 | -45.39 |
| 9-10 | - | - | - | - | - | - | - | - | - |
| Partially | 0.503 | 0.475 | 0.533 | 0.6906 | 0.6499 | 0.7338 | 30.94 | 26.62 | 35.01 |

| **Hospitalization** | **HR** | **HR 95% CI** | | **Adj IRR** | **Adj HR 95% CI** | | **Adj VE** | **Adj VE 95% CI** | |
|---|---|---|---|---|---|---|---|---|---|
| Vaccination status | | **low** | **up** | | **low** | **up** | | **low** | **up** |
| Fully vaccinated | 0.175 | 0.153 | 0.201 | 0.1168 | 0.1010 | 0.1350 | 88.32 | 86.50 | 89.90 |
| Time since vaccination, months | | | | | | | | | |
| 1-2 | 0.074 | 0.054 | 0.102 | 0.0543 | 0.0390 | 0.0755 | 94.57 | 92.45 | 96.10 |
| 3-4 | 0.210 | 0.172 | 0.257 | 0.1323 | 0.1072 | 0.1632 | 86.77 | 83.68 | 89.28 |
| 5-6 | 0.561 | 0.467 | 0.675 | 0.2614 | 0.2138 | 0.3196 | 73.86 | 68.04 | 78.62 |
| 7-8 | 1.465 | 1.175 | 1.825 | 0.7233 | 0.5655 | 0.9252 | 27.67 | 7.48 | 43.45 |
| 9-10 | - | - | - | - | - | - | - | - | - |
| Partially vaccinated | - | - | - | - | - | - | - | - | - |

| **Death** | **HR** | **HR 95% CI** | | **Adj IRR** | **Adj HR 95% CI** | | **Adj VE** | **Adj VE 95% CI** | |
|---|---|---|---|---|---|---|---|---|---|
| Vaccination status | | **low** | **up** | | **low** | **up** | | **low** | **up** |
| Fully vaccinated | 0.290 | 0.207 | 0.407 | 0.1409 | 0.0991 | 0.2002 | 85.91 | 79.98 | 90.09 |
| Time since vaccination, months | | | | | | | | | |
| 1-2 | 0.134 | 0.043 | 0.421 | 0.0763 | 0.0239 | 0.2434 | 92.37 | 75.66 | 97.61 |
| 3-4 | 0.4410 | 0.6735 | 0.2934 | 0.2156 | 0.1948 | 0.2287 | 79.49 | 77.16 | 81.74 |
| 5-6 | 1.644 | 1.384 | 1.924 | 0.3561 | 0.2301 | 0.4917 | 64.49 | 50.83 | 67.99 |
| 7-8 | 2.951 | 3.001 | 1.713 | 0.5153 | 0.4603 | 0.6518 | 48.47 | 34.82 | 53.97 |
| 9-10 | - | - | - | - | - | - | - | - | - |
| Partially vaccinated | - | - | - | - | - | - | - | - | - |

SI Table 5a. **mRNA vaccines**: COVID-19 infection, hospitalisation, death rates (per 100,000) due to **all variants in all ages**, by vaccination status and time since vaccination. Apulia region, Italy, Jan 1 – Dec 1, 2021

| Vaccination status | P/years infection | P/years hospitalization | P/years death | Infection N. | Hospitalization N. | Deaths N. | Infection rate | Hospitalization rate | Death rate |
|---|---|---|---|---|---|---|---|---|---|
| Fully vaccinated | 913,165.8 | 913,170.3 | 913,172.3 | 9,241 | 545 | 158 | 2.77 | 0.16 | 0.05 |
| Time since vaccination, months | | | | | | | | | |
| 1-2 | 333,796.7 | 333,801.2 | 333,798.5 | 2,487 | 124 | 43 | 2.04 | 0.10 | 0.04 |
| 3-4 | 331,170.6 | 331,173.8 | 331,172.3 | 3,030 | 127 | 36 | 2.51 | 0.11 | 0.03 |
| 5-6 | 185,508 | 185,510.3 | 185,509.6 | 2,305 | 175 | 50 | 3.4 | 0.26 | 0.07 |
| 7-8 | 53,556.02 | 53,557.15 | 53,557.55 | 1,096 | 112 | 29 | 5.61 | 0.57 | 0.15 |
| 9-10 | 9,073.627 | 9,073.707 | 9,073.627 | 319 | 7 | 0 | 9.63 | 0.21 | 0.00 |
| Partially vaccinated | 194,542.5 | 194,554.1 | 194,558.0 | 6,537 | 584 | 216 | 9.21 | 0.82 | 0.30 |
| Unvaccinated | 1,685,329.2 | 1,685,479.3 | 1,685,497.9 | 138,441 | 11,253 | 3,258 | 22.50 | 1.83 | 0.53 |
| Total | 2,793,037.5 | 2,793,203.7 | 2,793,226.9 | 154,206 | 12,382 | 3,632 | 4.96 | 1.21 | 0.36 |

SI. Table 5b. **Viral vector vaccines**: COVID-19 infection, hospitalisation, death rates (per 100,000) due to **all variants in all ages**, by vaccination status and time since vaccination. Apulia region, Italy, Jan 1 – Dec 1, 2021

| Vaccination status | P/years infection | P/years hospitalization | P/years death | Infection N. | Hospitalization N. | Deaths N. | Infection rate | Hospitalization rate | Death rate |
|---|---|---|---|---|---|---|---|---|---|
| Fully vaccinated | 172,326.26 | 172,326.99 | 172,326.5 | 2,829 | 86 | 6 | 4.50 | 0.14 | 0.01 |
| Time since vaccination, months | | | | | | | | | |
| 1-2 | 395,572.3 | 395,577.6 | 395,574.1 | 2,923 | 147 | 44 | 2.02 | 0.10 | 0.03 |
| 3-4 | 399,948.4 | 399,952.1 | 399,950.3 | 4,113 | 164 | 39 | 2.82 | 0.11 | 0.03 |
| 5-6 | 223,915 | 223,917.5 | 223,916.6 | 3,419 | 197 | 52 | 4.18 | 0.24 | 0.06 |
| 7-8 | 56,921.83 | 56,922.99 | 56,923.35 | 1,295 | 116 | 29 | 6.23 | 0.56 | 0.14 |
| 9-10 | 9,073.682 | 9,073.762 | 9,073.682 | 319 | 7 | 0 | 9.63 | 0.21 | 0.00 |
| Partially vaccinated | 65,310.38 | 65,311.53 | 65,311.5 | 1,249 | 75 | 20 | 5.24 | 0.31 | 0.08 |
| Unvaccinated | 1,685,329.19 | 1,685,479.31 | 1,685,482.3 | 138,428 | 11,253 | 3,258 | 22.50 | 1.83 | 0.53 |
| Total | 1,922,965.83 | 1,923,117.83 | 1,923,120.30 | 142,506 | 11,414 | 3,284 | 7.22 | 1.62 | 0.47 |

SI Table 6a: **mRNA vaccine**: Covid-19 infection, hospitalization, and death hazard ratios (HR) per 100.000 person days and adjusted VE in **all ages for all variants**, by vaccination status and time since vaccination. Apulia region, Italy, Jan 1 – Dec, 2021

| **Infection**<br>Vaccination status | **HR** | **HR 95% CI**<br>**low** | <br>**up** | **Adj IRR** | **Adj HR 95% CI**<br>**low** | <br>**up** | **Adj VE** | **Adj VE 95% CI**<br>**low** | <br>**up** |
|---|---|---|---|---|---|---|---|---|---|
| Fully vaccinated | 0.109 | 0.107 | 0.112 | 0.1090 | 0.1067 | 0.1114 | 89.10 | 88.86 | 89.33 |
| Time since vaccination, months | | | | | | | | | |
| 1-2 | 0.087 | 0.083 | 0.090 | 0.0859 | 0.0825 | 0.0895 | 91.41 | 91.05 | 91.75 |
| 3-4 | 0.105 | 0.101 | 0.109 | 0.1042 | 0.1004 | 0.1081 | 89.58 | 89.19 | 89.96 |
| 5-6 | 0.140 | 0.135 | 0.146 | 0.1458 | 0.1398 | 0.1520 | 85.42 | 84.80 | 86.02 |
| 7-8 | 0.228 | 0.214 | 0.242 | 0.2434 | 0.2291 | 0.2585 | 75.66 | 74.15 | 77.09 |
| 9-10 | 0.390 | 0.349 | 0.435 | 0.3809 | 0.3412 | 0.4253 | 61.91 | 57.47 | 65.88 |
| Partially | 0.350 | 0.341 | 0.360 | 0.3469 | 0.3379 | 0.3561 | 65.31 | 64.39 | 66.21 |

| **Hospitalization**<br>Vaccination status | **HR** | **HR 95% CI**<br>**low** | <br>**up** | **Adj IRR** | **Adj HR 95% CI**<br>**low** | <br>**up** | **Adj VE** | **Adj VE 95% CI**<br>**low** | <br>**up** |
|---|---|---|---|---|---|---|---|---|---|
| Fully vaccinated | 0.080 | 0.073 | 0.087 | 0.0636 | 0.0583 | 0.0693 | 93.64 | 93.07 | 94.17 |
| Time since vaccination, months | | | | | | | | | |
| 1-2 | 0.053 | 0.045 | 0.064 | 0.0538 | 0.0450 | 0.0643 | 94.62 | 93.57 | 95.50 |
| 3-4 | 0.054 | 0.045 | 0.065 | 0.0499 | 0.0418 | 0.0596 | 95.01 | 94.04 | 95.82 |
| 5-6 | 0.136 | 0.117 | 0.158 | 0.0879 | 0.0755 | 0.1023 | 91.21 | 89.77 | 92.45 |
| 7-8 | 0.307 | 0.254 | 0.370 | 0.1666 | 0.1379 | 0.2014 | 83.34 | 79.86 | 86.21 |
| 9-10 | 0.115 | 0.055 | 0.241 | 0.1375 | 0.0655 | 0.2887 | 86.25 | 71.13 | 93.45 |
| Partially vaccinated | 0.420 | 0.385 | 0.459 | 0.4471 | 0.4093 | 0.4884 | 55.29 | 51.16 | 59.07 |

| **Death**<br>Vaccination status | **HR** | **HR 95% CI**<br>**low** | <br>**up** | **Adj IRR** | **Adj HR 95% CI**<br>**low** | <br>**up** | **Adj VE** | **Adj VE 95% CI**<br>**low** | <br>**up** |
|---|---|---|---|---|---|---|---|---|---|
| Fully vaccinated | 0.078 | 0.067 | 0.092 | 0.0475 | 0.0404 | 0.0558 | 95.25 | 94.42 | 95.96 |
| Time since vaccination, months | | | | | | | | | |
| 1-2 | 0.077 | 0.057 | 0.105 | 0.0774 | 0.0570 | 0.1052 | 92.26 | 89.48 | 94.30 |
| 3-4 | 0.064 | 0.046 | 0.090 | 0.0568 | 0.0407 | 0.0792 | 94.32 | 92.08 | 95.93 |
| 5-6 | 0.173 | 0.130 | 0.230 | 0.0876 | 0.0659 | 0.1165 | 91.24 | 88.35 | 93.41 |
| 7-8 | 0.385 | 0.266 | 0.558 | 0.1345 | 0.0928 | 0.1950 | 86.55 | 80.50 | 90.72 |
| 9-10 | - | - | - | - | - | - | - | - | - |
| Partially vaccinated | 0.599 | 0.521 | 0.690 | 0.2708 | 0.1965 | 0.3561 | 72.92 | 64.39 | 80.35 |

SI Table 6b: Viral vector vaccines: Covid-19 infection, hospitalization, and death hazard ratios (HR) per 100.000 person days and adjusted in **all ages for all variants**, by vaccination status and time since vaccination. Apulia region, Italy, Jan 1 – Dec, 2021

| **Infection** | **HR** | **HR 95% CI** | | **Adj IRR** | **Adj HR 95% CI** | | **Adj VE** | **Adj VE 95% CI** | |
|---|---|---|---|---|---|---|---|---|---|
| Vaccination status | | **low** | **up** | | **low** | **up** | | **low** | **up** |
| Fully vaccinated | 0.167 | 0.161 | 0.174 | 0.1805 | 0.1738 | 0.1874 | 81.95 | 81.26 | 82.62 |
| Time since vaccination, months | | | | | | | | | |
| 1-2 | 0.082 | 0.075 | 0.090 | 0.0878 | 0.0798 | 0.0965 | 91.22 | 90.35 | 92.02 |
| 3-4 | 0.180 | 0.170 | 0.192 | 0.1932 | 0.1818 | 0.2052 | 80.68 | 79.48 | 81.82 |
| 5-6 | 0.327 | 0.308 | 0.347 | 0.3371 | 0.3176 | 0.3578 | 66.29 | 64.22 | 68.24 |
| 7-8 | 0.615 | 0.535 | 0.708 | 0.6198 | 0.5390 | 0.7127 | 38.02 | 28.73 | 46.10 |
| 9-10 | - | - | - | - | - | - | - | - | - |
| Partially | 0.313 | 0.305 | 0.320 | 0.3183 | 0.3107 | 0.3260 | 68.17 | 67.40 | 68.93 |

| **Hospitalization** | **HR** | **HR 95% CI** | | **Adj IRR** | **Adj HR 95% CI** | | **Adj VE** | **Adj VE 95% CI** | |
|---|---|---|---|---|---|---|---|---|---|
| Vaccination status | | **low** | **up** | | **low** | **up** | | **low** | **up** |
| Fully vaccinated | 0.063 | 0.051 | 0.078 | 0.0411 | 0.0332 | 0.0509 | 95.89 | 94.91 | 96.68 |
| Time since vaccination, months | | | | | | | | | |
| 1-2 | 0.053 | 0.035 | 0.080 | 0.0380 | 0.0252 | 0.0572 | 96.20 | 94.28 | 97.48 |
| 3-4 | 0.075 | 0.055 | 0.104 | 0.0539 | 0.0390 | 0.0746 | 94.61 | 92.54 | 96.10 |
| 5-6 | 0.083 | 0.055 | 0.126 | 0.0698 | 0.0459 | 0.1062 | 93.02 | 89.38 | 95.41 |
| 7-8 | 0.190 | 0.071 | 0.508 | 0.2140 | 0.0802 | 0.5710 | 78.60 | 42.90 | 91.98 |
| 9-10 | - | - | - | - | - | - | - | - | - |
| Partially vaccinated | 0.154 | 0.123 | 0.193 | 0.0999 | 0.0795 | 0.1256 | 90.01 | 87.44 | 92.05 |

| **Death** | **HR** | **HR 95% CI** | | **Adj IRR** | **Adj HR 95% CI** | | **Adj VE** | **Adj VE 95% CI** | |
|---|---|---|---|---|---|---|---|---|---|
| Vaccination status | | **low** | **up** | | **low** | **up** | | **low** | **up** |
| Fully vaccinated | 0.015 | 0.007 | 0.032 | 0.0083 | 0.0037 | 0.0186 | 99.17 | 98.14 | 99.63 |
| Time since vaccination, months | | | | | | | | | |
| 1-2 | 0.010 | 0.001 | 0.068 | 0.0073 | 0.0010 | 0.0519 | 99.27 | 94.81 | 99.90 |
| 3-4 | 0.025 | 0.008 | 0.078 | 0.0190 | 0.0061 | 0.0591 | 98.10 | 94.09 | 99.39 |
| 5-6 | 0.034 | 0.009 | 0.138 | 0.0370 | 0.0092 | 0.1483 | 96.30 | 85.17 | 99.08 |
| 7-8 | - | - | - | - | - | - | - | - | - |
| 9-10 | - | - | - | - | - | - | - | - | - |
| Partially vaccinated | 0.168 | 0.108 | 0.261 | 0.1072 | 0.0688 | 0.1670 | 89.28 | 83.30 | 93.12 |
